# Two new oribatid mites from the Republic of Rwanda. *Plasmobates zarae* sp. n. (Acari, Plasmobatidae) and *Basilobelba spasmenosi* sp. n. (Acari, Basilobelbidae)

**DOI:** 10.3897/zookeys.598.8972

**Published:** 2016-06-14

**Authors:** Nestor Fernandez, Pieter Theron

**Affiliations:** 1 National Council of Scientific and Technological Research (CONICET). Evolutive Genetic Laboratory FCEQyN, Misiones National University. Felix de Azara 1552, 6º, (3300) Posadas Misiones (Argentina); 2 Research Unit for Environmental Sciences and Management, North-West University, Potchefstroom Campus, 2520 (South Africa)

**Keywords:** Republic of Rwanda, Plasmobates zarae sp. n., Basilobelba spasmenosi sp. n., Afrotropic Ecozone

## Abstract

Two new species of oribatid mites, *Plasmobates zarae* **sp. n.** and *Basilobelba spasmenosi* **sp. n.** are described from the Republic of Rwanda. They can easily be differentiated from other species by a number of characters.

*Plasmobates zarae* **sp. n.** is differentiated the following characters. four types of particular cerotegumental layers. Integument slightly foveate to smooth on prodorsum; foveate on notogaster; ventral region rugose to smooth.Large rostral setae inserted on protuberance, whip-shaped, with longitudinal pucker; interlamellar setae rod-shaped with triangular scales; interlamellar setae small. Medial band on prodorsum extending to anterior of central part, but not reaching rostrum. Bothridium horn-shaped; opening basally incised with rectilinear wall, internal bothridial rings dentate. Sensillus whip-like, with minute triangular scales. Variably distributed circumgastric macropores. Opisthosomal gland apophysis flat, triangular in lateral view and cylindrical in posterolateral view. Six pairs of notogastral setae, all situated posterior to opisthosomal gland level. Aggenital setae not detected; three pairs of adanal setae; two pairs of anal setae present. Nymphal scalps simple without anterior tuft or filaments, with dentate peripheral ridge. Larval scalp shaped like Chinese hat.

*Basilobelba spasmenosi* **sp. n.** is characterized by the combination of the following characters: Cerotegument: thick basal layer with amorphous coat and cavities of different sizes, as well as structures resembling small cauliflowers. Setation: *simple*: notogastral, epimeral, genital, anal; *simple long*, *basally barbate*: *le*, *ro* setae; *simple*, *whip-shaped*: *ex* setae; *medium length*, *sharpened tip with thorns on surface*: *in* setae, leg setae; *Flabellate*: setae situated in ventral neotrichous zone. Thorn-like barbs and more or less parallel longitudinal grooves present on body surface of *le*, *ro*, *in* and leg setae. Prodorsum: rostrum finger-shaped, relative sizes of setae: *le* > *ro* > *in* > *ex.* Prodorsal cuticular surface smooth with shallow transversal furrow and two oblique furrows determining two triangular structures. Large humpbacked *CSO* situated anterior to and in medial line with *in* setal insertion, dorsal bothridial opening. Notogaster swollen, hemispheric; nine pairs of minute setae, only *h_1_*, *h_2_*, *h*3 easily identifiable, cuticular wart and dimple clearly visible. Humeral apophysis with longitudinal furrow dorsally. Elongate chelicera with *cha*, *chb* setae, behind them a series of scales directed dorsoventrally. Epimeral setation 3-1-3-3, adanal-aggenital neotrichy with between 8-10 setae. Nymphal scalps with very particular bean-shaped structure on either side of the decoupage zone, surrounding horn-like structure. Scalps with cuticular polyhedral reticulate to ovoid structure, often forming a cavity, either completely perforated or with a thin cuticular layer resembling an interior membrane.

## Introduction

This paper is the second on material collected in Rwanda, housed at the Natural History Museum in Geneva, Switzerland. Two new species, *Plasmobates zarae* sp. n. and *Basilobelba spasmenosi* sp. n. are described. At present the family Plasmobatidae consists of four genera: *Orbiculobates* Grandjean, 1961; *Malgachebates* Fernández, Cleva, Theron, 2011; *Plasmobates* Grandjean, 1929 and *Solenozetes* Grandjean, 1931. Over the course of many years the authors have studied members of the family Plasmobatidae, principally those collected from the Afrotropic ecozone (formerly known as Ethiopian zone). This resulted in the description of the genus *Malgachebates* and included a summary of the principal characteristics of each genus of the family (Fernandez et al. 2011). Fernandez et al. (2013) analyzed some problematic aspects of the genus *Solenozetes* and presented a redefinition of the genus, as well as the description of *Solenozetes makokouensis* Fernandez et al., 2013.

The taxonomy of the family Plasmobatidae is problematic due to succinct original descriptions lacking in detail, or in which important characteristics were neglected.

*Plasmobates* (*sensu* Subías 2015) consists of the following species: *Plasmobates pagoda* Grandjean, 1929, *Plasmobates carboneli* Pérez-Íñigo & Sarasola, 1998, *Plasmobates hyalinus* Hammer, 1971, *Plasmobates asiaticus* Aoki, 1973, *Plasmobates africanus* Balogh, 1958 “sp. inq.”, *Plasmobates foveolatus* Ermilov, Sidorchuk & Rybalov, 2010, *Plasmobates machadoi* Balogh, 1958 “sp. inq.”, *Plasmobates minor* Balogh, 1958 “sp. inq.”. The last four species are from the Afrotropic ecozone, and three of the four species are “species inquerendae” (*sensu* Subías 2015) (see Discussion). We continue the study of this group by providing a description of *Plasmobates zarae* sp. n. Despite more than forty years in alcohol, material was in an excellent state of preservation, conserved to the point that adequate SEM studies could be conducted.

The family Basilobelbidae contains two genera: *Basilobelba* Balogh, 1958 and *Xiphobelba* Csiszár, 1961. The taxonomy of Basilobelbidae is not clear, principally relating to the problematic original description of *Xiphobelba* (Csiszar 1961 page: 353) which indicates: “Rostrum pointed, chelicerae attenuated to a point, chela very small reduced” and “The new genus is an ally of *Basilobelba* Bal.1958, differing from it by the peculiar oral organs, resembling those of *Eupelops*”. The oral organs were not illustrated, and other characteristics, such as the cerotegumental layer were neither described nor figured. Only two figures were given, one dorsal with scalps and the other dorsal without scalps. In the description of a second species of the genus, *Xiphobelba setosa* Aoki, 1968, the chelicera are partly illustrated with the rest of the infracapitulum (Aoki 1968 p: 271, figure 14). Due to several subsequent papers, the taxonomy of *Basilobelba* is becoming clearer, permitting understanding of several aspects of this group.

More recently Fernandez et al. (2015) described a new species of *Basilobelba* (*Basilobelba maidililae*) from Vietnam, analysed problematic aspects of the group, and provided a comparison of species of both genera of the family.

## Materials and methods

Specimens studied by means of light microscopy were macerated in lactic acid, and observed in the same medium using the open-mount technique (cavity slide and cover slip) as described by Grandjean (1949) and Krantz and Walter (2009). Drawings were made using a Zeiss GFL (Germany) compound microscope equipped with a drawing tube.

Specimens were also studied under a Scanning Electron Microscope (SEM). Specimens preserved in ethanol were carefully rinsed by sucking them several times into a Pasteur pipette, after which they were transferred to buffered glutaraldehyde (2.5%) in Sörensen phosphate buffer: pH 7.4; 0.1 m for two hours. After postfixation for 2hr. in buffered 2% OsO_4_ solution and being rinsed in buffer solution; all specimens were dehydrated in a series of graded ethanol and dried in a critical point apparatus. After mounting on Al-stubs with double sided sticky tape, specimens were gold coated in a sputter apparatus (Alberti and Fernandez 1988, 1990a, 1990b; Alberti et al. 1991, 1997, 2007; Fernandez et al. 1991). SEM micrographs were taken using a SEM FEI-Quanta Feg 250; with 10 Kv and working distance (WD) variable.

Measurements: total length (tip of rostrum to posterior edge of notogaster); width (widest part of notogaster) in micrometers (μm). Leg setation was studied using standard, polarized and phase contrast microscopes are provisional, due to the fact that only adult specimens were available for study. Setal formulae of the legs include the number of solenidia (in parentheses); tarsal setal formulae include the famulus (ε). For *Plasmobates zarae* we added SEM images of leg setae as detail in order to clarify a number of particularities.

### Morphological terminology

Morphological terms and abbreviations used herein are those developed by F. Grandjean (1928–1974) (cf. Travé and Vachon 1975). For the setae types Evans (1992); ornamentation of cuticular surfaces Murley (1951, *ex*: Evans *op.cit*) were used. Some specific morphological characters have never been described before in detail, and hence no terminology or abbreviations exist. For the sake of clarity we include the following in the text and on the figures: bean-shaped structure (h.sc); macropores (mp); medial band extension (m.b); polyhedral reticulate to ovoid structure (s.r.s); promontories of podocephalic canal (a.o.g); thin cuticular layer (t.c.l).

## New taxa description

### Plasmobates zarae sp. n.

Taxon classificationAnimaliaSarcoptiformesPlasmobatidae

http://zoobank.org/4D90B90C-D50E-4465-B125-128295332B6F

Figures 1–8 , 9–13 , 14–19 , 20–24 , 25–33 , 34–42 , Table 1

#### Etymology.

The specific epithet “*zarae*” is derived from (ζάρα, Grec=pucker, English) due to longitudinal pucker present on *ro* setae.

#### Material examined.

Holotype: Female and two paratypes (adult females): “73/2. Kayove-Rwanda; 2100 mts. 15/V/1973” Leg. P.Werner; deposited in the Collection of the Natural History Museum of Geneva (M.H.N.G), Switzerland; preserved in 70% ethanol. Material studied for SEM: three specimens, not deposited.

#### Diagnosis (adult female).

Cerotegumental layer. *Amorphous*: bothridial zone, tubercle of seta *in*, *ro* setae insertion, lateral gland, epimeral zone, genital plate and surrounding zone, anal plate and surrounding zone. *Layer with small tubercles*: internal bothridial zone. *Mixed-layer* (mushroom-like microtubercles associated with irregular cauliflower-like microtubercles): infracapitulum, epimeral zone, lateral body zone, basal zone lateral gland. Integument: prodorsum, small foveate to smooth; notogaster, foveate; ventral region rugose to smooth.

Setation: *simple*: lamellar, notogastral, exostigmatal, epimeral, genital, aggenital anal; *whip-shaped*, *with longitudinal pucker*: rostral setae; *rod-shaped with triangular scales*: interlamellar setae; *simple*, *basally inflated*: subcapitular *a*; *simple spur*: *m*.

Prodorsum: medial band extension on central part towards anterior, not extending to rostrum. Interlamellar setae inserted on large protuberances, lamellar setae small, rostral setae large, with longitudinal cuticular puckers, inserted on protuberances. Large horn-shaped bothridium, directing laterally, rectilinear wall with basally incised opening. Internal bothridial rings dentate. Whip-shaped sensillus with minute, triangular scales; exostigmatal seta small. Rostrum medially incised, posterior of incision rounded. Notogaster: fovea situated in smooth zone with circumgastrically distributed macropores on fovea margins or inside fovea. Opisthosomal gland apophysis flat, triangular in lateral view, cylindrical in posterolateral view. Six pairs of notogastral setae, all situated posterior to level of opistosomal gland. Lateral region: opening of podocephalic canal on large promontories.

Ventral region: epimeral setal formula (3-1-2-2). Seven pairs of genital setae; aggenital setae not detected. Three pairs of adanal setae, two pairs of anal setae. Scalps multilayered, medial band extending anteriorly from each scalp. Medial band covers central zone, firmly adhered to prodorsal surface. Nymphal scalps with dentate peripheral ridge. Setae hardly discernible, scalps simple without anterior tuft of filaments. Chinese hat-shaped larval scalp differing greatly from nymphal scalps

#### Description.

**Figures 1–8.**
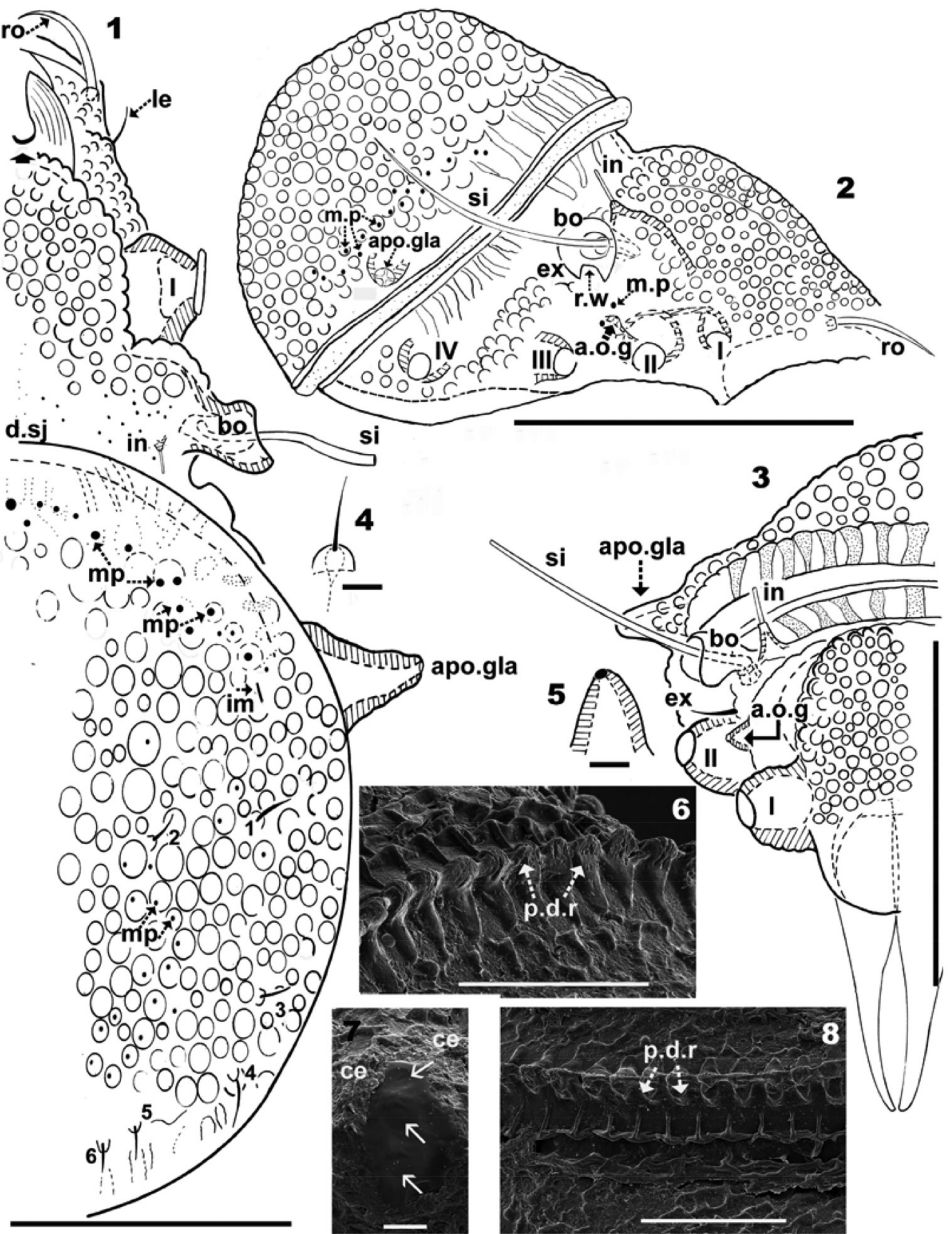
*Plasmobates zarae* sp. n. Adult; **1–5** optical microscopy image **6–8** SEM **1** dorsal view **2** lateral view **3** frontal view **4** lamellar setae **5** promontories podocephalic canal **6** dentate peripheral ridge (*p.d.r*), lateral inclined view **7** cerotegumental layer and cuticular microsculpture **8** dentate peripheral ridge (*p.d.r*), frontal view. Abbreviations: see Materials and methods. Scale: **1** = 100 μm; **2** = 200 μm; **3** = 70 μm; **8** = 50 μm; **6** = 30 μm; **7** = 10 μm; **4, 5** = 5 μm.

**Figures 9–13.**
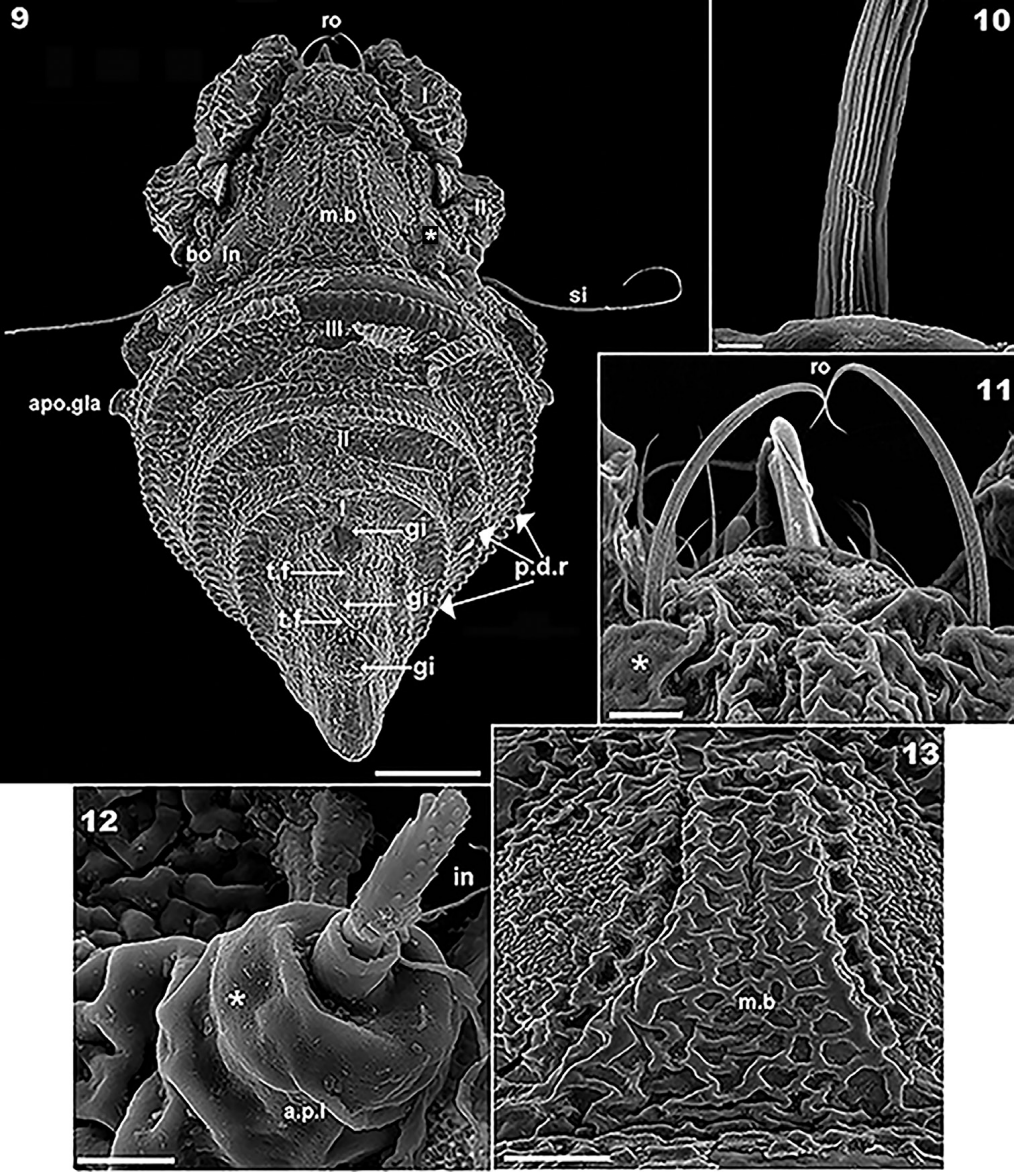
*Plasmobates zarae* sp. n. Adult; SEM micrographs. **9** dorsal view with scalp and cerotegument layer **10** rostral setae, detail **11** rostral setae, general view **12** interlamellar seta **13** medial band detail, dorsal view. Abbreviations: see Materials and methods. Scale: **9** = 100 μm; **11, 13** = 20 μm; **12** = 5 μm; **10** = 2 μm.

**Figures 14–19.**
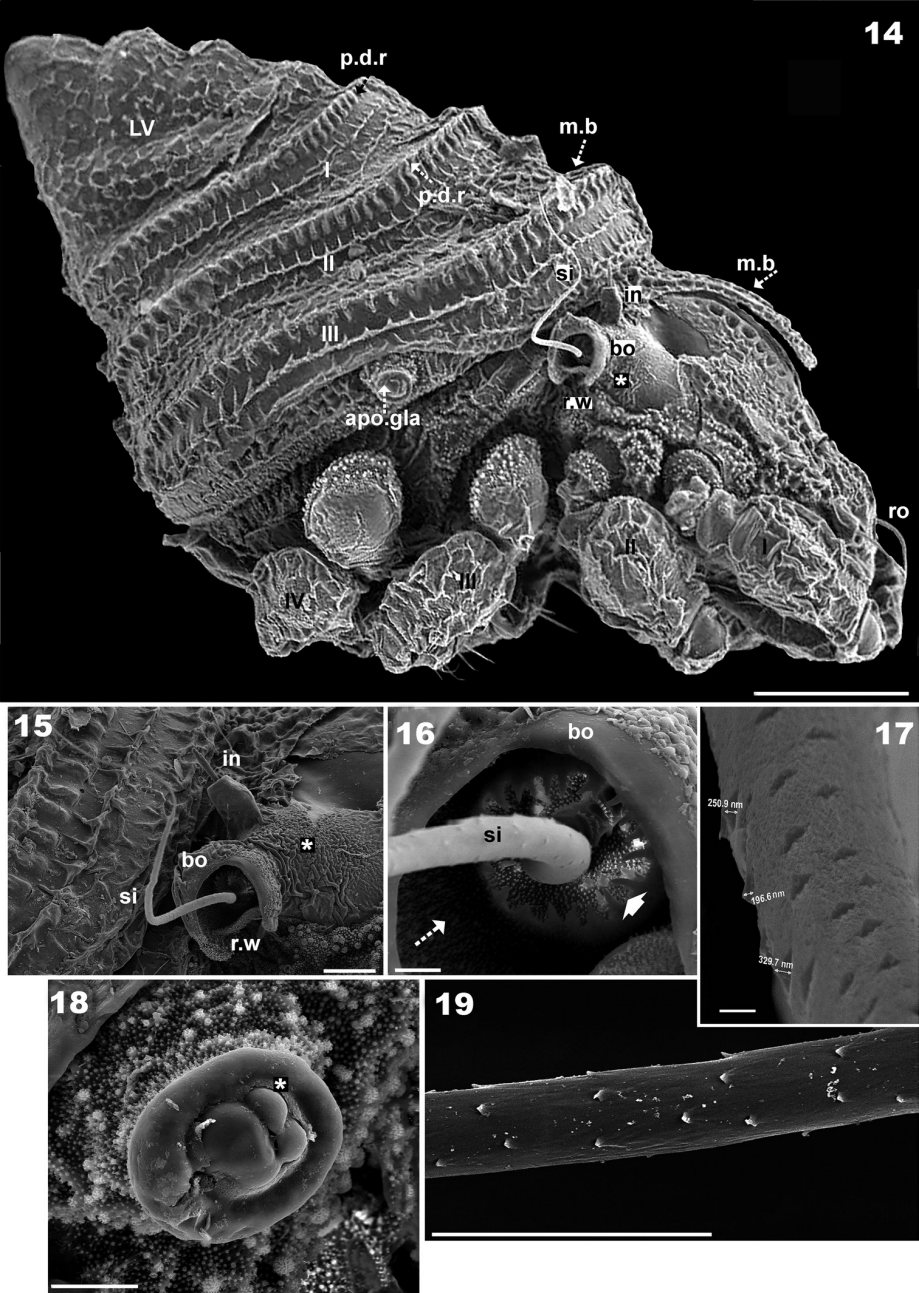
*Plasmobates zarae* sp. n. Adult; SEM. **14** lateral view with scalp and cerotegumental layer **15** bothridium and interlamellar seta, lateral view **16** bothridium, internal structures **17** sensillus, superficial scales (high magnification) **18** lateral gland **19** sensillus detail. Abbreviations: See Materials and methods. Scale: **14** = 100 μm; **15** = 20 μm; **18** = 10 μm; **16, 19** = 5 μm; **17** = 1 μm.

*Measurements.* SEM: total length with scalps 580–615 μm × 600 μm (measurements on three specimens). Total length without scalps 433–438 μm × 435μm (measurements on three specimens). Notogastral width without scalps 248–253 μm × 250 μm. Light microscopy: 612–656 μm × 639 μm (measurements on three specimens). Specimens with scalps ovoid, elongate in dorsal view. (Figures 1, 9, 22). In lateral view specimens with scalps appear pyramidal (Figure 14); without scalps anterior triangular and posterior rounded (Figure 2).

**Figures 20–24.**
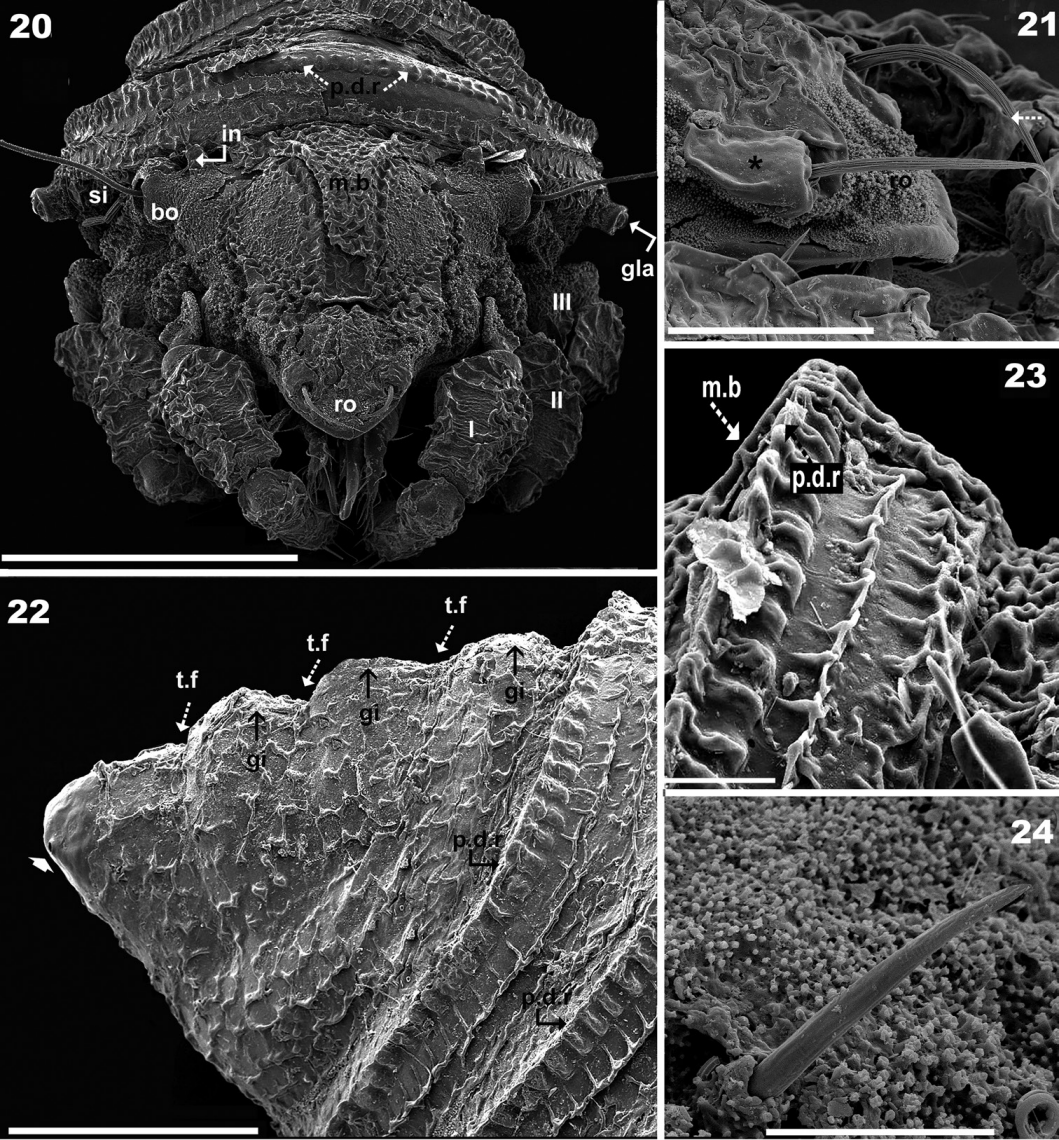
*Plasmobates zarae* sp. n. Adult; SEM. **20** frontal view **21** *ro* seta **22** tritonymphal scalp with medial bands, lateral view **23** larval scalp detail **24** *ag* seta detail. Abbreviations: see Materials and methods. Scale: **20** = 200 μm; **22** = 20 μm; **23** = 50 μm; **21** = 10 μm; **24** = 5 μm.

*Colour*. Specimens without cerotegument and scalps dark yellowish to medium brown.

**Figures 25–33.**
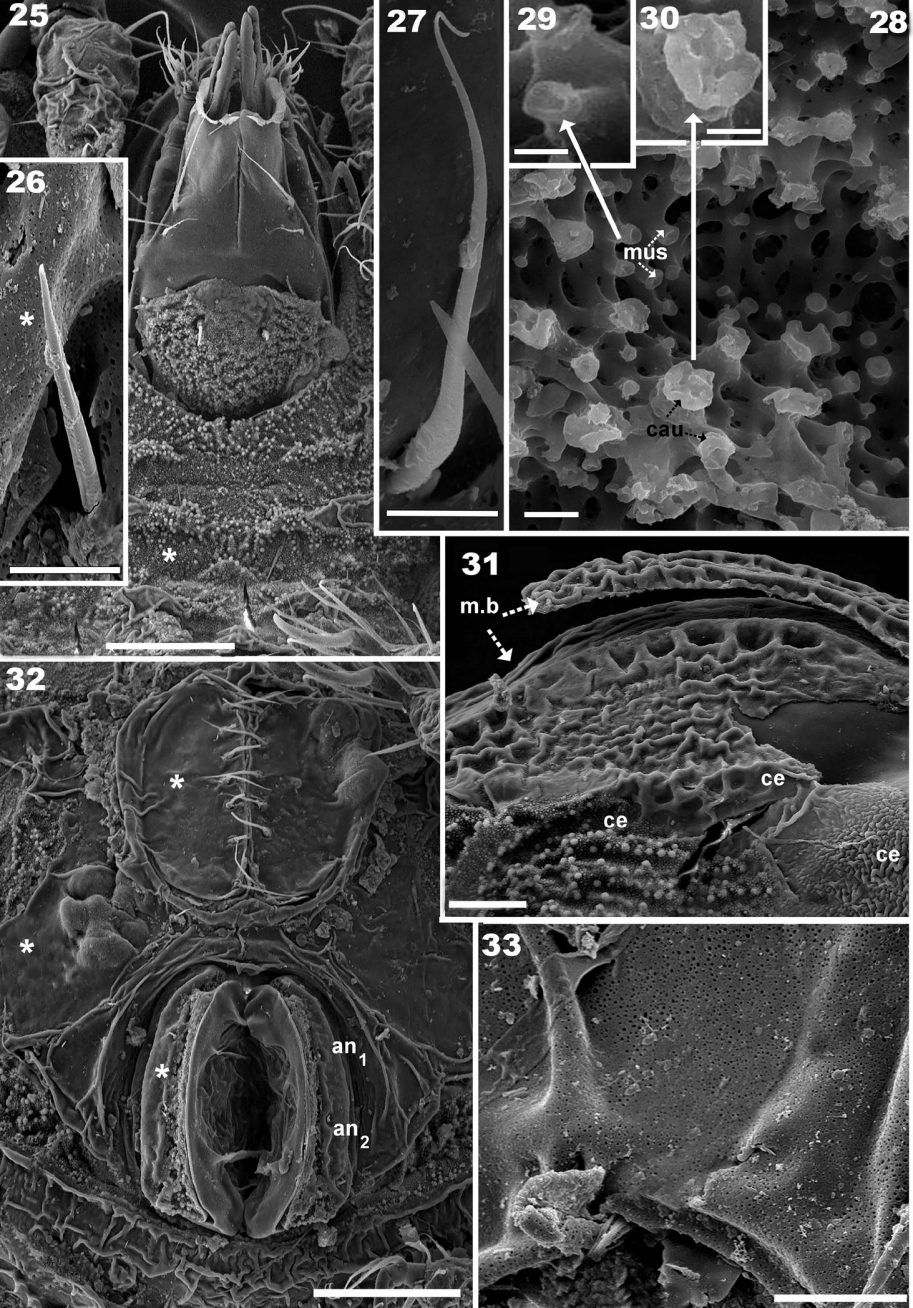
*Plasmobates zarae* sp. n. Adult; SEM. **25** epimeral zone **26** subcapitular seta *h* **27** subcapitular seta *a* **28** cerotegumental layer **29** detail cerotegumental “cauliflower” (*cau*) **30** detail cerotegumental “mushroom” (*mus*) **31** prodorsum with *m.b*. **32** anogenital region **33** detail cerotegumental layer. Abbreviations: see Materials and methods. Scale: **25** = 50 μm; **26** = 5 μm; **27** = 5 μm; **28** = 1 μm; **29** = 0.5 μm; **30** = 0.5 μm; **31** = 20 μm; **32** = 50 μm; **33** = 5 μm.

*Cerotegument* (scalps not considered). Thick complex layer with elaborate pattern, composed of wax layer and amorphous cement layer covering entire body and legs. *Amorphous layer* (Figure 33, indicated in all Figures with *): external bothridial zone of prodorsum (Figures 14, 15), tubercles of *in* setae (Figure 12), *ro* setae insertion zone (Figure 21), *gla* (Figure 18), epimeral zone (Figure 25) subcapitular setae *h* (Figure 26), genital plate and surrounding zone, anal plate and surrounding zone (Figure 32). *Small tubercules*: internal bothridial zone (Figure 16 indicated by5). *Mixed-layer* (Figures 28, 29, 30): mushroom-like microtubercles (*mus*) diameter 0.02–0.6 μm, height 0.2–1.9 μm associated with irregular cauliflower-like microtubercles (*cau*) diameter 1.2–1.9 μm, height 1.4–3.1 μm. Distribution: infracapitulum, epimeral zone, lateral body zone, basal zone of *gla* (Figures 18, 19, 25, 31). Legs: Trochanters covered by *mixed-layer*, femur, genu, tibia, covered by *amorphous layer* with prominent folds. Tarsus: *amorphous layer* with subtle folding and several smooth areas (Figures 14, 20, 35, 36, 38, 39, 42).

**Figures 34–42.**
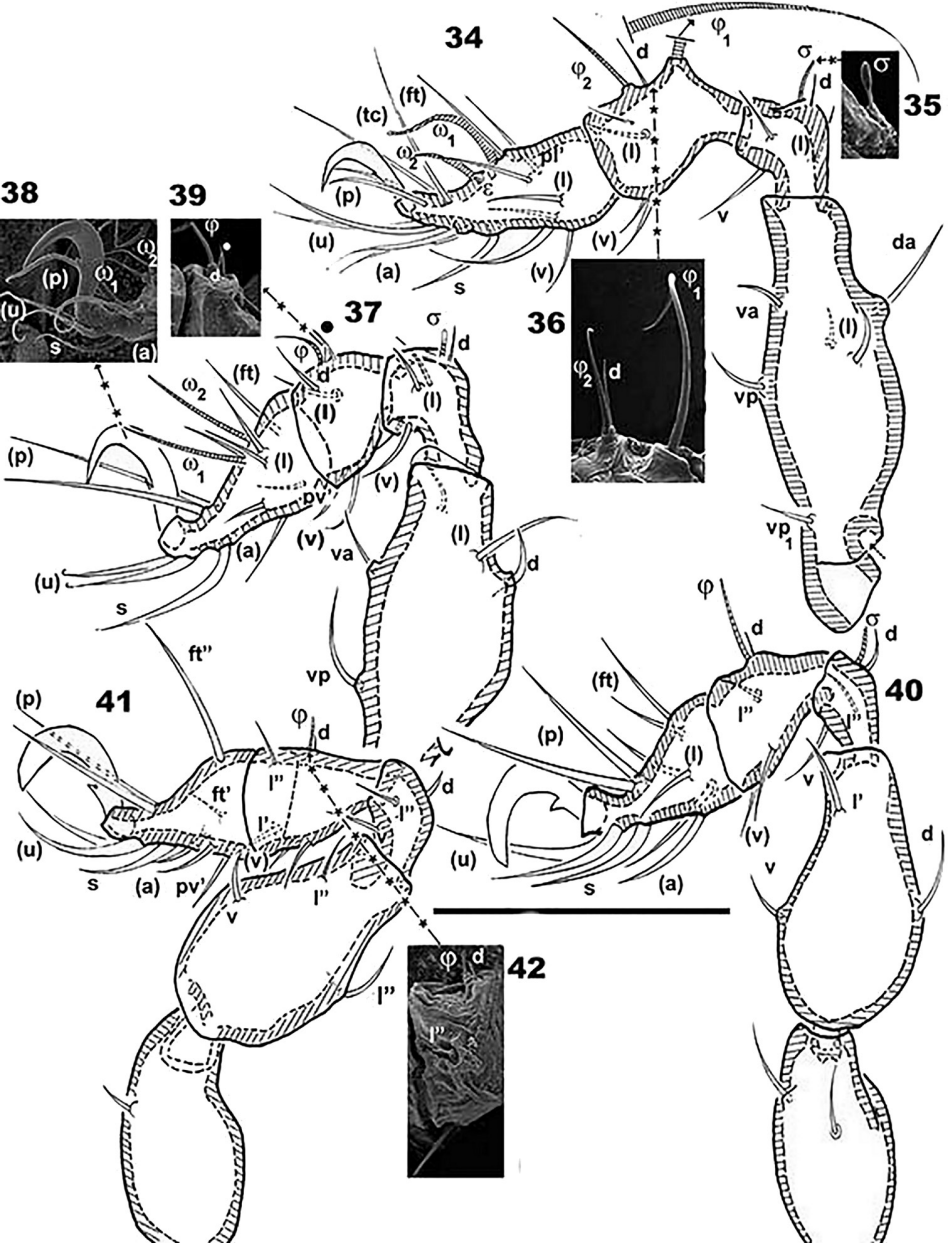
*Plasmobates zarae* sp. n. Adult; Optical and SEM observations. **34** leg I, antiaxial **35** detail with SEM; solenidium s genu I **36** detail with SEM, solenidia j_1_, j_2_ and setae *d*, femur I **37** leg II, antiaxial **38** apical zone, tarsus II (detail with SEM) **39** solenidium j_1_, dorsal setae *d* and complementary seta indicated byl, tibia I (detail SEM observation) **40** leg III, antiaxial **41** leg IV, antiaxial **42** tibia I, solenidium j and setae *d* (detail SEM observation). Abbreviations: see Materials and methods. Scale: **34–42** = 70 μm.

*Integument.* lateral microsculpture of prodorsum faintly foveate to smooth (Figures 1, 2, 3), dorsal microsculpture of prodorsum flat, foveate (Figure 7 indicated by h). Notogaster: foveate in vicinity of notogastral border (Figures 1, 2, 3), posterior notogastral zone smooth, posterolateral notogastral zone presenting ridges anterior to macropore zone (Figures 1, 2, 3). Distribution of macropores circumgastric (Figures 1, 2). Ventral region rugose to smooth on subcapitulum (Figure 25), epimeral zone, surrounding genital and anal openings and genital and anal plates.

*Setation.* Lamellar (Figure 4), notogastral, exostigmatal, epimeric, genital, aggenital (Figure 24) and anal setae simple; *ro* setae whip-shaped, with longitudinal pucker (Figures 9, 10, 11); *in* setae (10–13 μm) rod-shaped with triangular scales (Figure 12); subcapitular setae simple, slightly basally inflated (Figure 27) 30–36 μm *a*; *h* simple, spur-shaped (Figure 26) 16–21 μm.

*Prodorsum.* Medial band extension (*m.b*) observed on central part towards anterior, not extending to rostrum, terminating anterior to *le* setal insertion level on specimens with scalps (Figures 9, 13, 14, 20, 31). Elevated zone surrounding medial band extension (Figure 14). Interlamellar setae (*in*) inserted near bothridial base on large protuberances, extending upward and inclined backward (Figures 9, 12, 14, 15). Lamellar setae (*le*) small, inserted on small protuberances (Figure 4), rostral setae (*ro*) (58-61 μm) inserted on protuberances, cuticular folds at base of setae (Figures 10, 11, 14, 20, 21). Large laterally directing horn-shaped bothridium (Figures 14, 20). Semicircular lateral bothridial opening, basally incised with thin rectilinear wall (*r.w*) (Figures 14, 15). Internal bothridial rings dentate with triangular teeth (Figure 16).

Whip-shaped filiform sensillus (*si*) (80-106 μm) with minute triangular scales, height 196 nm, length 603-987 nm (Figure 17), exostigmatal setae (*ex*) small. Narrow medial incision on rostrum, in dorsal view posterior end of incision rounded (Figure 1).

*Notogaster.* Circumgastrically distributed macropores (*mp*) of varying diameter (0.3–1 μm) situated in small foveae on smooth zone, either on periphery or internally to foveated notogastral pattern (Figures 1, 2). In dorsal view anterior zone *mp* clearly visible (Figure 1), but those located near *gla* need to be observed in lateral or posterior views (Figure 2) due to notogastral shape, in order to obtain the best impression of their distribution.

Distribution of *mp*: a) single line in anterior notogastral zone; b) linear in anterior lateral zone near *gla*; c) irregularly distributed on posterior notogastral zone (setal zone) (Figure 1).

In dorsal view opisthosomal gland (*gla*) apophysis observed as flat triangle, but appears cylindrical in lateral and lateroposterior views, directing slightly obliquely forward (Figures 1, 9, 14, 18); opening with protuberances (Figure 18). Six notogastral setae on small protuberances (Figure 1), all setae situated behind level of *apo.gla*.

*Lateral region.* Exobothridial seta (*ex*) small but clearly discernible (Figure 3); two macropores situated one above and one below promontories of podocephalic canal (Figure 2 surrounding *a.o.g*). Opening of podocephalic canal on large promontories (Figures 2, 3, 5). Tubercles of interlamellar setae more or less cylindrical (Figures 12, 15); setae *ro* inserted on tubercles (Figures 12, 14); notogastral border clearly discernible even after long preservation in lactic acid; sejugal depression deep, easily discernible.

*Ventral region.* Specimens with cerotegument: plate-like cerotegumental structures on epimeres resulting in irregular levels on upper surface, epimeral furrows easily discernible with *mus*, *cau* and amorphous cerotegumental layer (Figure 25). Flat lateral cerotegumental zones, deep epimeral furrows 1 and 2. Epimeres III, IV small, epimeral setal formula (3-1-2-2). Seven pairs of genital setae in a single longitudinal line; aggenital setae not detected; three pairs of adanal setae; two pairs of anal setae.

*Gnathosoma.* Subcapitulum suctorial with short tube. Subcapitular setae large, especially *a*, *m* (Figure 25).

*Legs* (Table 1). Legs differ from those of congeners studied by the authors (See Table 1), particularities illustrated on SEM micrographs (Figures 34–42). Setal and solenidial formulae (trochanter to tarsus): I(1-6-4-5-19-1) (1-2-2); II(1-4-5-6-12-1) (1-1-2); III(2-3-3-4-11-1) (1-1-0); IV(1-3-4-5-10-1) (0-1-0).

**Table 1.** *Plasmobates zarae* sp. n. adult, legs.

	Femur	Genu	Tibia	Tarsus	Observations
**Leg I**					
Setae	*da*,(*l*),*va*,*vp*, *vp_1_*	(*l*),*v*,*d*	*d*,(*l*),(*v*)	*pl*’,(*l*),(*v*),(*ft*),(*tc*),(*p*),(*u*),(*a*),*S*,(*v*),e	Crispinate (socket-like) dorsal femur, solenidium s clavate, *d* seta positioned near j_2_, usually near j_1_
Solenidium	-	s	j_1_, j_2_	w_1_, w_2_	
**Leg II**					
Setae	*l*”,*d*,*va*,*vp*	*d*,(*l*),(*v*)	*d*,l,(*l*)(*v*)	(*ft*),(*l*),(*p*)(*u*),*S*,(*a*),*pv*’	crispinate (socket-like) dorsal femur, solenidium s clavate, genu with one *d* seta near j, also another associated minute seta indicated by l
Solenidium		s	j	w_1_, w_2_	
**Leg III**					
Setae	*d*,*l*’,*v*,	*d*,*l*”, *v*	*d*,*l*”,(*v*)	(*ft*),(*p*),(*u*),*S*,(*a*),(*l*)	
Solenidion		s	j	-----------------	
**Leg IV**					
Setae	(*l*),*v*	*d*,*l*”,*v*	*d*,(*l*),(*l*)	(*ft*),*pv*’,(*a*),*S*,(*u*),(*p*)	
Solenidion		---------	j	---------------------	

*Scalps.* Exuviae of immature stases adhering one on top of the other, creating a multilayered structure. Each scalp extending anteriorly into a medial band (*m.b*) (Figures 14, 20, 23, 31) covering central zone, adhering to prodorsal surface (Figure 20) and extending backward towards *ro* setal insertion (Figure 20). Sometimes *m.b* is slightly detached (Figure 14).

Cerotegumental layer: medial band covered by thick amorphous layer with a network of round to polygonal depressions (Figure 13). Nymphal scalps with dentate peripheral ridge (*p.d.r*) (Figures 6, 8, 14, 20, 22, 23). Setae hardly discernible, scalps simple without anterior tuft of filaments. Larval scalp unlike the others, broad and elevated, Chinese hat-shaped, with three gibbose areas (*gi*) separated by transverse furrows (*t.f*) (Figure 22). In lateral view insertion of *dp* setae clearly visible (Indicated by Figure 22).

#### Remarks.

SEM is vital in order to observe aspects such as: 1) dorsal seta *d* associated with solenidium hardly discernible (detailed drawings are included to facilitate understanding) 2) clavate shape of small solenidia is problematic, as doubt regarding the exact shape remains if using only optical microscopy 3) the position of dorsal seta *d* relative to j_1_ and j_2_ on tibia II differs from *Solenozetes makokouensis* and *Malgachebates peyrierasi*. Changed angles of observation and rotation of specimens in SEM clarified the situation. 4) accessories available in SEM facilitated measurements of minute triangular scales of the sensillus with great precision 5) protuberances situated around the opening of the lateral gland also had to be observed from different angles. See Discussion for comparison with congeners.

### Basilobelba spasmenosi sp. n.

Taxon classificationAnimaliaSarcoptiformesBasilobelbidae

http://zoobank.org/F4ABFDAF-99F0-45DB-876C-DD914C7852A7

Figures 43–47 , 48–52 , 53–57 , 58–62 , 63–69 , 70–74 ; Table 2

#### Etymology.

The specific epithet “*spasmenosi*” is derived from (Σπασμένος, Grec = broken, English), due to characteristics of scalps with cavities or perforations.

#### Material examined.

Holotype: Female and two paratypes (adult females):. “73/2. Kayove-Rwanda; 2100 mts.15/V/1973” Leg. P.Werner; material deposited in the Collection of the Natural History Museum of Geneva (M.H.N.G), Switzerland; preserved in 70% ethanol. Material studied for SEM: three specimens, not deposited.

#### Diagnosis (adult female).

Cerotegument. Thick basal layer with amorphous coat, perforations of various sizes, and structures resembling small cauliflowers. Setation. *Simple*: notogastral, epimeral, genital, anal; *simple*, *long*, *basal barbs*: *le*, *ro* setae; *simple*, *whip-shaped*: *ex* setae; *medium length*, *sharpened tip with thorn-like barbs on surface*: *in* setae, leg setae; *flabellate*: setae situated in ventral neotrichy zone. Thorn-like barbs and more or less parallel longitudinal grooves on body of *le*, *ro*, *in* and leg setae. Prodorsum. Rostrum finger-shaped, relative sizes of setae: *le* > *ro* > *in* > *ex.* Prodorsal cuticular surface smooth, with shallow transversal furrow and two oblique furrows delineating two triangular structures. Large humpbacked *CSO* situated anterior to and in medial line with *in* setal insertion, dorsal bothridial opening. Notogaster. Swollen, hemispheric, with nine pairs of minute setae. Only *h_1_*, *h_2_*, *h*_3_ easily identifiable. Cuticular wart and dimple clearly visible. Humeral apophysis with longitudinal furrow dorsally. Chelicera elongate, series of dorsoventrally directing scales behind setae *cha*, *chb.* Epimeral setation 3-1-3-3, adanal-aggenital neotrichy with 8-10 setae. Nymphal Scalps. Particular bean-shaped structure on either side of the decoupage zone around horn-like structure. Scalps with polyhedral reticulate to ovoid cuticular structure. Polyhedral reticulate cuticular structure often appears either completely perforated or with a thin cuticular layer resembling an interior membrane.

#### Description.

*Measurements.* SEM: total length without scalps 618–598 × 605 μm; width without scalps 310–290 × 303 μm (measurements on three specimens). Light microscopy: 660–632μm × 643 μm; width 325–315 × 320 μm (measurements on three specimens).

**Figures 43–47.**
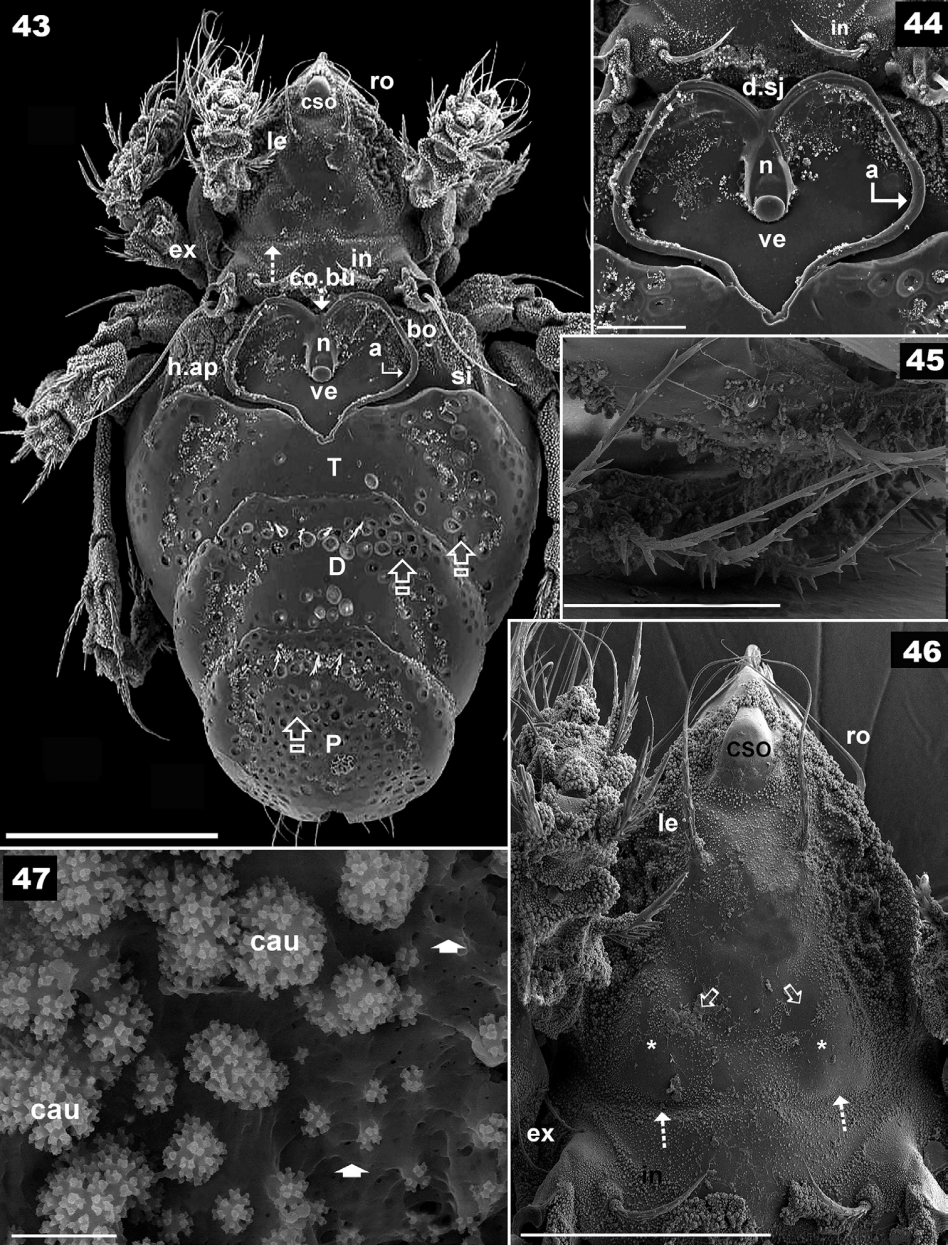
*Basilobelba spasmenosi* sp. n. Adult; SEM. **43** dorsal view with scalps **44** dorsal view with scalp **45** posterior zone of scalps **46** prodorsum anterior zone **47** arched tritonymphal buckle. Abbreviations: see Materials and Methods. Scale bars: **43** = 200 μm; **44** = 50 μm; **45** = 20 μm; **46** = 50 μm; **47** = 2 μm.

*Shape.* Elongated oval (Figures 43, 49, 53).

**Figures 48–52.**
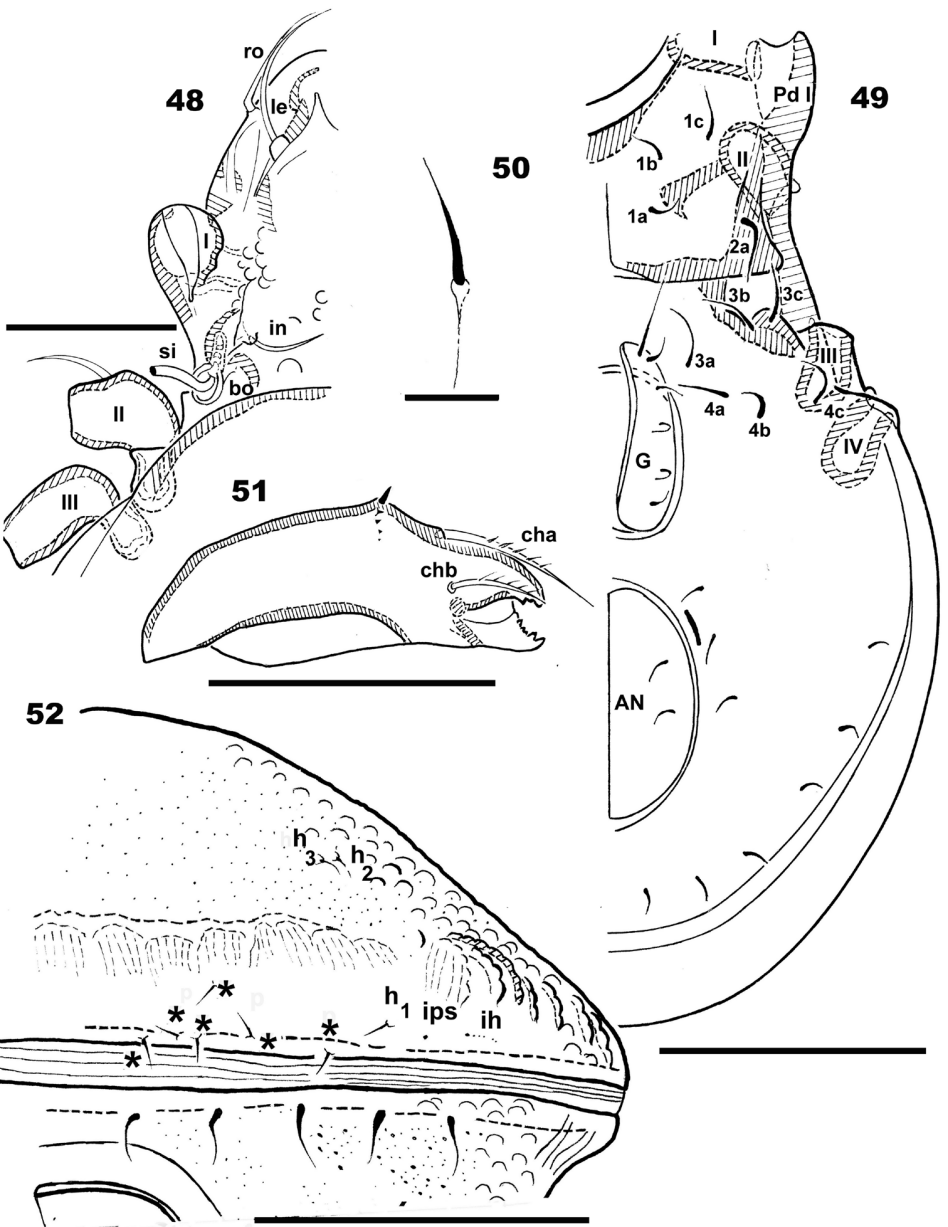
*Basilobelba spasmenosi* sp. n. Adult; Optical microscopy. **48** anterior zone prodorsum **49** ventral zone without subcapitulum **50** ventral setae **51** Chelicera, lateral view **52** notogaster posterior view. Abbreviations: see Materials and methods. Scale bars: **52** = 100 μm; **49** = 70 μm; **50** = 25 μm; **48, 51** = 20 μm.

**Figures 53–57.**
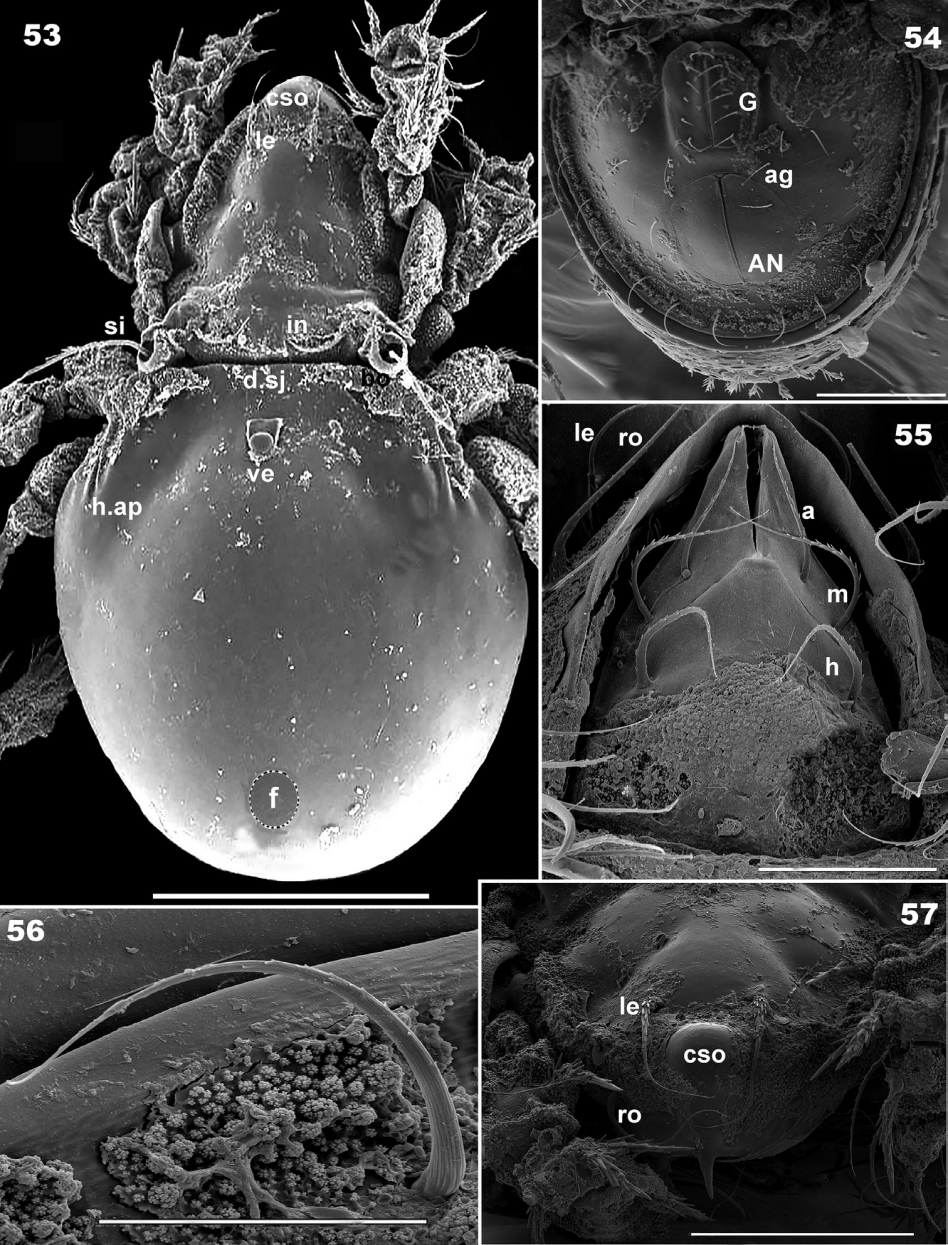
*Basilobelba spasmenosi* sp. n. Adult; SEM. **53** general view without scalps **54** genito-anal zone **55** subcapitulum, ventral view **56** ventral setae **57** frontal view. Abbreviations: see Materials and methods. Scale bars: **53** = 200 μm; **54** = 100 μm; **55** = 20 μm; **56** = 20 μm; **57** = 100 μm.

*Colour.* Specimens without cerotegument brown, slightly shiny when observed in reflected light.

*Cerotegument.* Present only on prodorsum, notogastral anterior zone, ventral region and legs. Thick basal layer with amorphous coat and perforations of various sizes (indicated by¿ Figure 47). Small structures on surface resembling cauliflowers (*cau*) of different sizes (Figures 47, 56, 59, 60).

**Figures 58–62.**
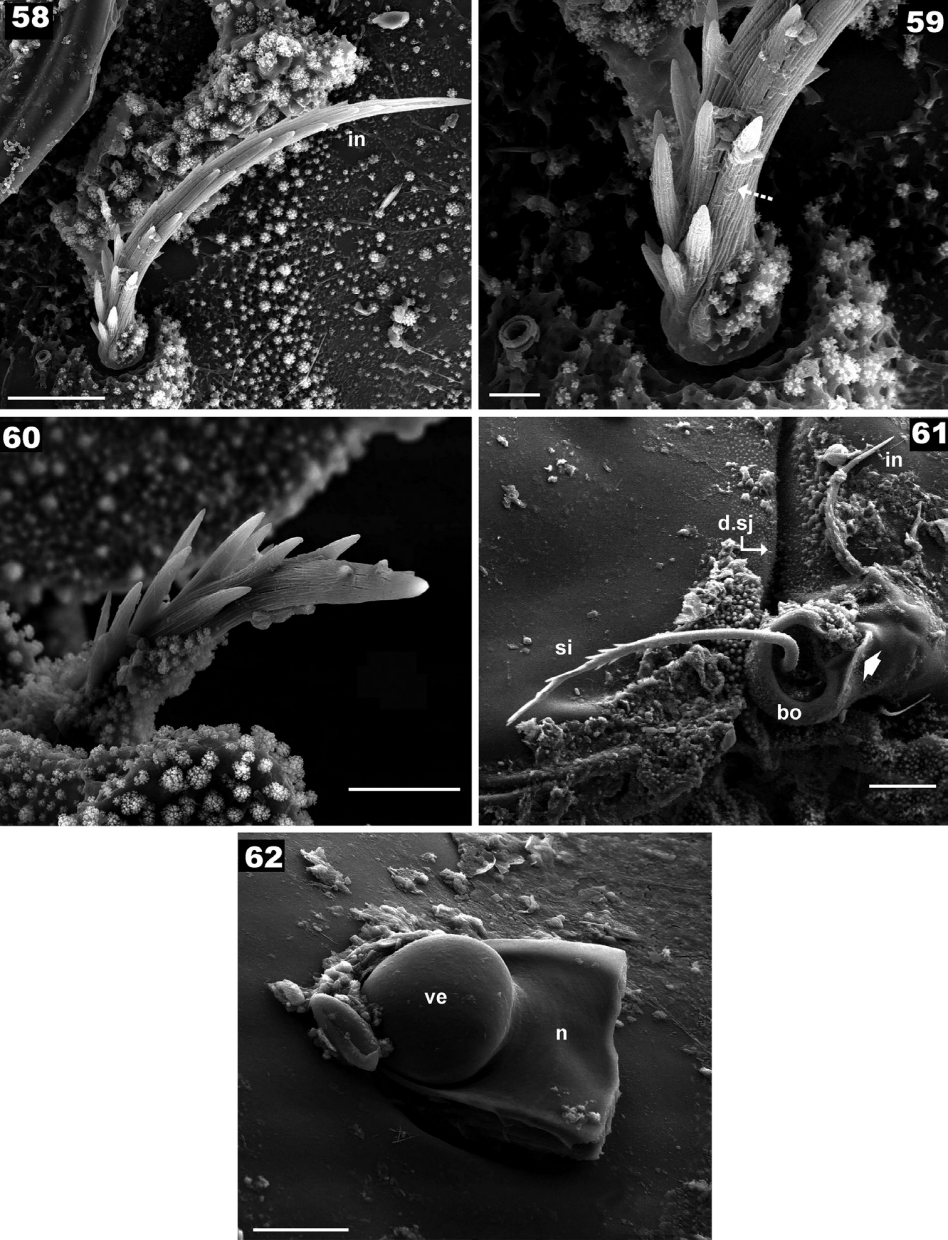
*Basilobelba spasmenosi* sp. n. Adult; SEM. **58** *in* seta **59** detail *in* seta **60** legs, dorsal setae **61** bothridium **62** wart, with part of tritonymphal buckle. Abbreviations: see Materials and methods. Scale bars: **58** = 10 μm; **59** = 2 μm; **60** = 10 μm; **61** = 20 μm; **62** = 10 μm.

*Setation. Simple*: notogastral, epimeral, genital, anal (Figures 50, 52, 54); *simple*, *long*, *basal barbs*: *le*, *ro* setae (Figures 48, 55); *simple*, *whip-shaped*: *ex* setae (Figures 43, 46); *medium length*, *sharpened tip with thorns on surface*: *in* setae (Figures 43, 44, 46, 58, 59, 61), leg setae. These setae are very particular, with large thorn-like barbs basally and small thorn-like barbs distally. *Flabellate* (Figure 56): setae situated in ventral neotrichous zone. Particular to *le*, *ro*, *in* and leg setae (Figure 60) is the presence of thorn-like barbs and more or less parallel longitudinal grooves on setal body (Figures 58, 59, 60).

*Integument.* Smooth

*Prodorsum.* Rostrum finger-shaped (Figures 43, 46). Rostral setae *ro* laterally inserted on large promontories 107 μm (96–109 μm); *le* setae 155 μm (151–159 μm); *in* setae on small promontories 46 μm (48–50 μm); *ex* 40 μm (38–42 μm). Relative sizes of setae: *le* > *ro* > *in* > *ex*.

Prodorsal cuticular surface smooth with a shallow transversal furrow situated anterior to *in* setal insertion (Figure 46 indicated by 5). Two oblique furrows (Figure 46 indicated by a) delimiting two triangular structures (Figure 46 indicated by *). Large humpbacked *CSO* situated in front of and in medial line with *in* setal insertion (Figures 43, 46, 53). More or less parallel *le* setae with criss-crossing tips (Figure 46). Two oblique furrows and two triangular structures conspicuous in dorsal view of prodorsum.

Ovoid bothridial opening dorsally (Figures 43, 48, 53); in medial zone ovoid loop directing anteriorly and slightly obliquely (Figure 61 indicated by¿). Sensillus long, setiform, generally directing backward, both sides barbate (Figure 61).

*Frontal view.* Beak-shaped rostral margin (Figure 57). Large humpbacked *CSO* clearly visible, slightly anterior to interlamelar setal insertion.

*Notogaster.* Swollen, hemispheric (Figure 53), bearing four-layered exuviae (larval, protonymphal, deutonymphal and tritonymphal) stacked to resemble a low tower (Figure 43), fixed anteriorly and posteriorly by particular structures (See Scalps). Dorsosejugal furrow large, rectilinear, well delimited (Figure 44). After removal of exuviae, glabrous notogastral surface becomes visible (Figure 53), bearing nine pairs of minute setae (Figure 52). Only *h_1_*, *h_2_*, *h_3_* setae easily identifiable (*h_2_* and *h_3_* always in very close proximity to each other), *h*_1_ setae identified by relative position to lyrifissures (See Discussion). Unidentified setae indicated by * (Figure 52). Lyrifissures *ih* and *ips* identified as pores, other lyrifissures probably present but difficult to identify due to ornamentation. Anterior notogastral zone (Figures 43, 44) bearing cuticular wart (*ve*) hooking arched tritonymphal buckle (*co.bu*) by coaptation. Depression in posterior zone (Figure 53, dimple (*f*) indicated by rounded dotted zone) housing the *us* zone of tritonymphal horn by coaptation. (Figure 69 indicated by *us* and ¿). SEM observations of this small depression is necessary from different angles, hence it is indicated by a rounded zone (See Scalps). Humeral apophysis easily discernible, with dorsal longitudinal furrow (Figure 43).

*Lateral region.* Only pedotectum I present (Figure 49); *Pd I* large lamina; the border can be followed a short distance to *ex* setae; *h.ap* clearly discernible as a structure with longitudinal furrow (Figure 43).

*Ventral region.* Subcapitulum diarthric, cerotegumental layer observed only behind *h* setal insertions (Figure 55). Subcapitular setae faintly barbate on either side, sharply tipped. Setae differing greatly in shape (see Figure 55): *a* (42 μm ± 3 μm) simple, sharply tipped; *m* (55 μm ± 3 μm); *h* (48μm ± 3 μm). Chelicera (Figure 51) elongate, with *cha*, *chb* setae. Series of dorsoventrally directing scales posterior to *cha*, *chb* setae; the largest is found dorsally, appears darker, followed by small transparent scales. Small movable digit (Figure 51) (see Discussion).

Epimeres I, II typical morphology, ventrosejugal furrow easily discernible, other epimeres not visible. Epimeral setation 3-1-3-3 (Figures 49, 54). Aggenital and adanal setae difficult to identify due to adanal-aggenital neotrichy. Neotrichy originates laterally to anal opening, is very prominent on posterior zone, number of setae varies between 8-10 (See Discussion).

*Legs* (Figures 70–74, Table 2). Leg shape similar to *Basilobelba retiarius* (Grandjean, 1959), moniliform with bulbous segments and large peduncles (Figures 70–74), femoral peduncles being largest. Tarsi particularly shaped, narrower between bulb and claw on legs I-IV. Cerotegumental layer covering segments but only basal zone of setae (Figure 60).

**Figures 63–69.**
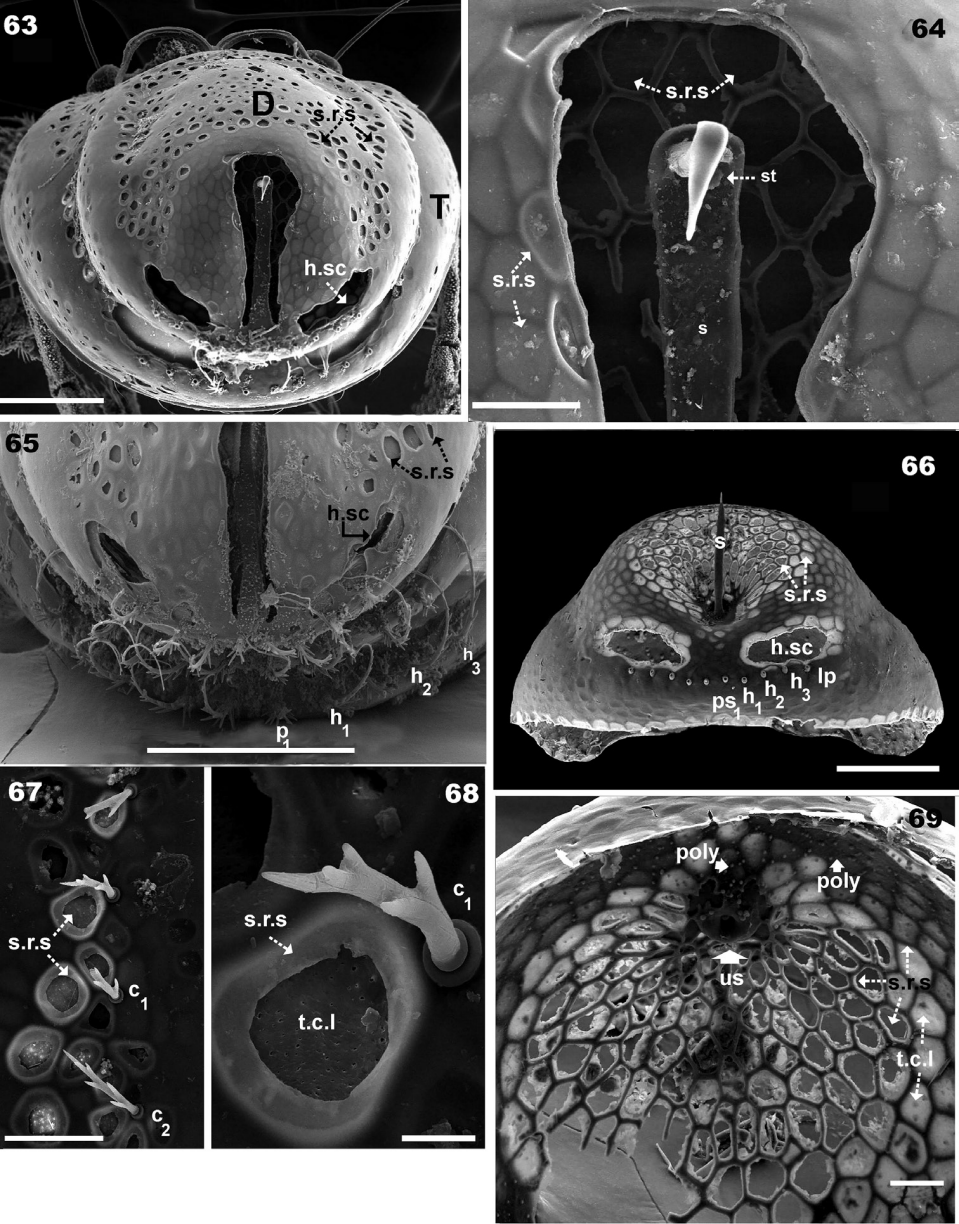
*Basilobelba spasmenosi* sp. n. Adult; SEM. **63** posterior view deutonymphal and tritonymphal scalps **64** posterior zone deutonymphal scalps, detail **65** dorso-posterior view deuto and tritonymphal scalps **66** tritonymphal scalps, posterior view **67** deutonymphal scalp, detail, anterior zone **68** detail *s.r.s* and *c1* seta, deutonymphal scalp **69** interior view, tritonymphal scalp. Abbreviations: see Materials and methods. Scale bars: **63** = 100 μm; **64** = 20 μm; **65** = 50 μm; **66** = 100 μm; **67** = 20 μm; **68** =5 μm; **69** = 20 μm.

**Figures 70–74.**
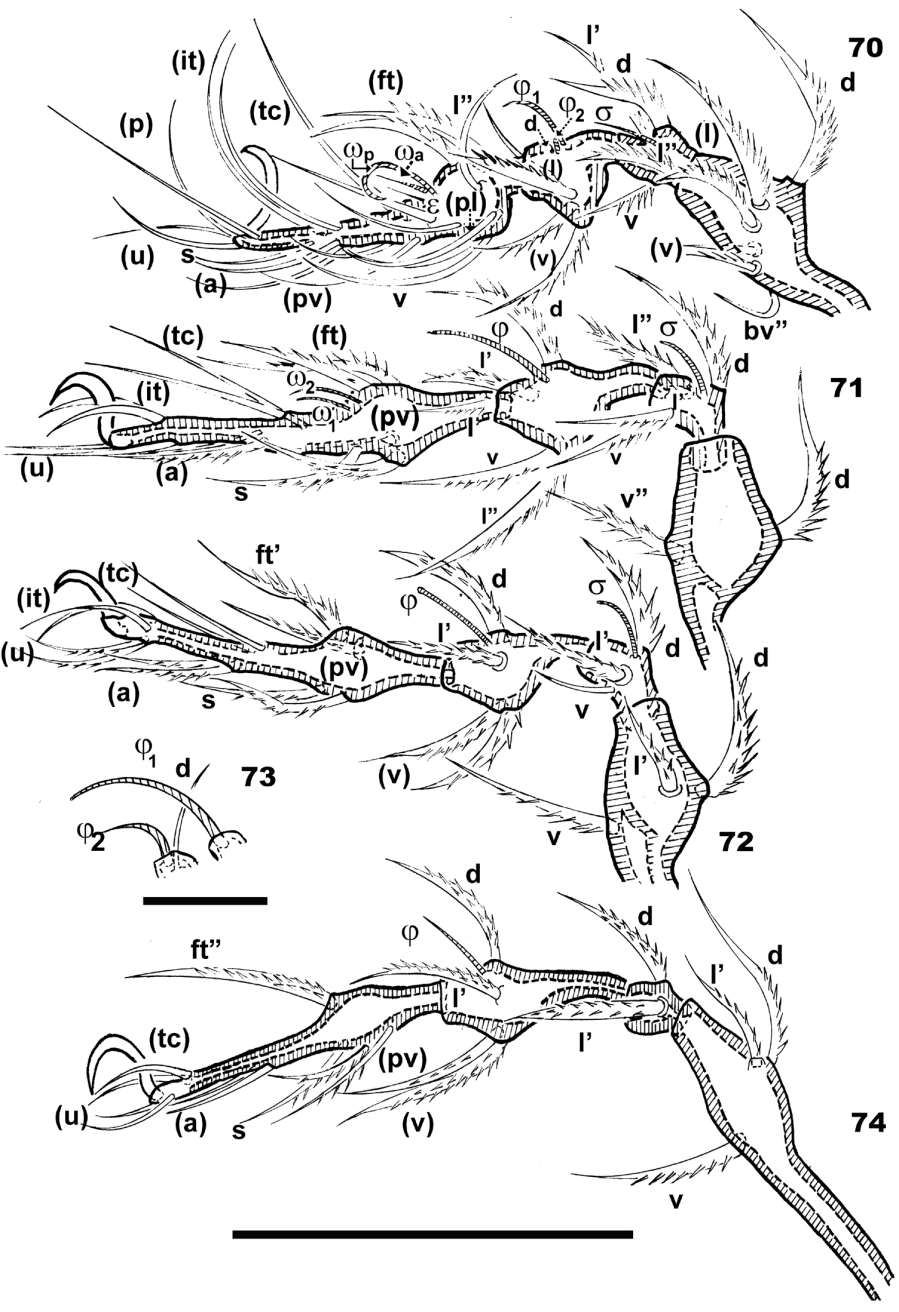
*Basilobelba spasmenosi* sp. n. Adult; Optical observations. **70** leg I antiaxial **71** leg II antiaxial **72** leg III antiaxial **73** details tibia I solenidium j1, j2 and dorsal seta **74** leg IV, antiaxial. Abbreviations: See Materials and methods. Scale bars: **70, 71, 72, 74** = 100 μm; **73** = 25 μm.

**Table 2.** *Basilobelba spasmenosi* sp. n. adult, legs.

	Femur	Genu	Tibia	Tarsus
Leg I				
Setae	*d*,(*l*),(*v*),*bv*	(*l*),*d*,*v*	*d*,(*l*),(*v*)	(*ft*),*l*”,(*pl*),*v*,(*pv*),(*tc*),(*it*),(*p*),(*u*),*s*,(*a*),*e*
Solenidium	--------	s	j_1_ , j _2_	w_1_, w_2_
Leg II				
Setae	d,v”	d,(l),v	d,(l),v	(*ft*),(*pv*),*S*,(*a*),(*u*),(*it*),(*tc*),*l*’
Solenidium		s	j	w_1_, w_2_
Leg III				
Setae	d,l’,v	d,v,l’	d,l’(v)	*ft*’,(*pv*),(*tc*),(*it*,)(*u*),(*a*),*S*
Solenidium				
Leg IV				
Setae	d,l’,v	d,l’	d,l’(v)	(*tc*),*ft*”,(*pv*)*s*,(*a*)(*u*)
Solenidium		-----	j	-------

Setal formulae: I(1-6-4-5-20-1) (1-2-2); II(1-2-4-4-14-1) (1-1-2); III(2- 3-3-4-12-1) (1-1-0); IV(1-3-2-4-10-1) (0-1-0). Setae *d* present on all femurs, genua and tibiae. On tibia I (Figure 70) seta *d* is small and hardly discernible (Figure 73), situated on the same promontory as solenidion φ_2_. On all other tibiae (II, III, IV) (Figures 71, 72, 74),genua and femurs setae *d* large and barbate (Figures 70, 71, 72, 74).

*Nymphal Scalps.* Limited number of specimens and the necessity of dissection impeded comprehensive study of scalps, for this reason our study was limited to deutonymphal and tritonymphal scalps. Observed particularities: Very particular bean-shaped structures are found on either side of the decoupage zone around the horn-like structure (Figures 63, 64, 65, 66).

Scalps present polyhedral reticulate to ovoid cuticular structure (*s.r.s*), most visible on internal side (Figure 69) but also on entire scalp (Figures 63–69). Internally scalps present a very thin cuticular layer (*t.c.l*) (Figure 69) covering polygonal-ovoid structures. The *s.r.s* often appearing either completely perforated or with a thin cuticular layer (*t.c.l*) resembling an interior membrane (Figures 63, 65, 66, 67). In Figure 64 the *s.r.s* is clearly visible due to transparency, also internally, on both sides of the decoupage zone around the horn. The complexity of these perforated areas is yet more interesting as the polyhedral reticular structure, when not perforated, is more or less rounded, surrounded by a polyhedral structure (Figure 67, 68).

*Tritonymphal scalps* (Figures 43, 45, 66, 69). Basque beret-shaped (Figure 66) tritonymphal scalp fixed to the adult by two structures, one situated anteriorly and the other posteriorly. Heart-shaped structure (tritonymphal buckle) in anterior part affixing scalp to adult notogaster. Tritonymphal buckle consisting of two loops (*a*) (Figures 43, 44), curving outwards then inwards forming a heart-shaped structure, continuing to meet in the plane of symmetry forming a thong-like structure (*n*) (Figure 44) in order to receive the wart (*ve*) (Figures 53). The *ve* is a round-ovoid structure (Figure 62) situated on the adult cuticular surface, functioning like a snap button, fixing the anterior part of the scalp to the adult cuticle. Depression (*f*) on the posterior adult cuticular surface (Figure 53) is indicated by a dotted round zone with *f* in centre. Observing *f* is difficult, necessitating changes in angle of observation. Zone *f* functions by coaptation with the interior part (*su*) (Figure 69 indicated by ¿) (i.e. the inner curving part of the horn-like structure). Small polyhedral structures (poly) are present (Figure 69 indicated by poly and¿) with similar characteristics to *Basilobelba maidililae* Fernandez et al., 2015 but obtaining high resolution SEM images was impossible due to a technical problem. Setae *h_1_*, *h_2_*, *h_3_*, *p_1_* clearly visible; setae *lp*, *lm* only visible in some instances, due to the cerotegumental layer impeding observation (Figure 65). Horn-like structure on posterior scalp border (Figure 64, 65) aiding in hooking the deutonymphal scalp.

*Deutonymphal scalps* (Figures 43, 63, 64, 65). Tritonymphal and deutonymphal scalps differ greatly (Figures 66 and 63). No buckles for adherence observed in anterior zone of deutonymphal scalps, and posterior zone (Figures 63, 64) with horn-like structure fixing the protonynphal scalp found on a mobile strip (*le*) consisting of a section of deutonymphal scalp (See Grandjean 1959). Horn-like structure composed of a style (*s*) and a stylet (*st*) (Figure 64). In common with the tritonymphal scalp, a bean-shaped structure (*h.sc*) is observed. Only posterior setae *h_1_*, *h_2_*, *h_3_*, *p_1_* are visible.

#### Remarks.

The remarkable perforated structures are not observed in other congeners. At present we are studying another species from Rwanda with bean-shaped structures on scalps, similar to those in *Basilobelba spasmenosi* sp. n.

## Discussion

The taxonomy of the genus *Plasmobates* in the Afrotropic ecozone is very complex. Species of the genus *Plasmobates* Grandjean, 1929 recorded in this region are: *Plasmobates africanus* Balogh, 1958; *Plasmobates foveolatus* Ermilov et al., 2010; *Plasmobates machadoi* Balogh, 1958; *Plasmobates minor* Balogh, 1958; and *Plasmobates zarae* sp. n. Subías (2015) considers *Plasmobates africanus* Balogh, 1958; *Plasmobates machadoi* Balogh, 1958 and *Plasmobates minor* Balogh, 1958 as “sp. inq.”, without providing reasons. We analyzed the work of Balogh (1958), but found it impossible to identify the cited species. A comprehensive search in the available collection from this ecozone failed to provide specimens with the characters in the provided text. One comparable species from the region is *Plasmobates foveolatus* Ermilov et al., 2010, but the study lacks SEM micrographs, and is not detailed enough to provide a conclusive comparison.

In our opinion the following characteristics permit easy differentiation of species of *Plasmobates* from other congeners, not only those from the Afrotropic ecozone: the cerotegumental layer, shape and insertion type of *ro* and *in* setae, sensillus with scales, promontories of podocephalic canal, distribution of macropores, type of lateral gland, number and distribution of notogastral setae, positions of setae *d* of tibia I and particular setae found on tibia II.

The taxonomy of *Basilobelba* Balogh, 1958 and *Xiphobelba* Csiszár, 1961 were discussed in preceding work (Fernandez et al. 2015). *Basilobelba spasmenosi* sp. n. presents all characteristics of the genus and also displays very interesting particularities, permitting easy differentiation from other congeners, such as: shape and characteristics of cerotegumental layer, lamellar and rostral setae, prodorsum, rostrum and *CSO*, infracapitulum, notogastral setae, nymphal scalps with perforated areas and particular distribution of leg setae. The distribution of notogastral setae is especially particular as all nine setae are situated on the posterior notogastral zone.

## Supplementary Material

XML Treatment for Plasmobates zarae

XML Treatment for Basilobelba spasmenosi
